# “No reason for children to go hungry”: Rural U.S. caregivers’ perceptions of elected officials and their impacts on food policy

**DOI:** 10.1371/journal.pone.0320453

**Published:** 2025-07-22

**Authors:** Annie Hardison-Moody, Lindsey Haynes-Maslow, Sarah K. Bowen

**Affiliations:** 1 Department of Agricultural and Human Sciences, North Carolina State University, Raleigh, North Carolina, United States of America; 2 Department of Health Policy and Management, UNC Gillings School of Global Public Health, Chapel Hill, North Carolina, United States of America; 3 Department of Sociology and Anthropology, North Carolina State University, Raleigh, North Carolina, United States of America; Paris School of Business, FRANCE

## Abstract

In the context of the increasingly polarized U.S. political environments of the 2016 and 2020 elections, how did rural caregivers think about food and health policies? To answer this question, researchers interviewed 50 low-income women living in two rural N.C. counties in 2016/7 and 2020 as part of a broader longitudinal qualitative study of family food environments. As participants reflected on elections and food assistance policies, caregivers focused on their experiences as mothers and described current or potential policies and programs in light of how they would impact their children. Caregivers also made recommendations for universal and inclusive policies that would improve their access to food. However, their responses were also racialized, with White caregivers more likely to use tropes of “deservingness” when discussing public benefits. To improve food security in rural areas, researchers should center rural low-income women’s perspectives when developing food policy, particularly given the important role mothers play in feeding their families and mitigating the impacts of food insecurity.

## Introduction

Rural U.S. Americans experience worse health outcomes than their urban counterparts, with higher rates of suicide [1], food insecurity [2], and diet-related chronic disease [3]. These outcomes are related to multiple factors, including inconsistent access to healthcare, a lack of grocery stores [4] safe places to be active [5], persistent poverty [6], and continued economic disinvestment in rural areas. Since the 1990s, many rural communities have experienced declines in farming and manufacturing sectors [7], combined with rising unemployment and underemployment, and youth outmigration [8]. These economic factors, as well as the closure and consolidation of hospitals and healthcare centers, have created a “rural health crisis” that was only exacerbated by the COVID-19 pandemic [1,9].

Researchers have adopted the framework of “deaths of despair” to show how rises in rural morbidity are tied to economic insecurity and a reduction in social investments in rural areas [10]. Researchers also note that negative health outcomes are not evenly distributed across rural places or people. For example, rural people of color have lower life expectancies and experience higher health-related morbidity than their White counterparts [11]. Researchers have also noted the lack of attention to the perspectives, priorities, and experiences of rural individuals, women, in particular [9].

One strand of research on rural health disparities has looked at food access and food insecurity. The places with the highest rates of food insecurity in the United States are disproportionately rural, 15.4% of households in non-metropolitan areas were classified as food insecure, compared to 13.2% in metropolitan areas [2]. Relatively few studies have explicitly focused on the lives and experiences of rural food-insecure families [12,13]. However, researchers argue that place matters when it comes to food and food policy. People living in rural areas often lack convenient and affordable access to grocery stores and face challenges accessing federal food and emergency food benefits due to a culture of stigmatization and shame. These experiences are shaped by their rural environments [12–14]. At the same time, rural residents also report stronger social ties, which could mitigate the physical and emotional impacts of food insecurity [12]. There is a need for additional research on how these contextual realities shape rural food insecurity. Furthermore, existing research has considered how access to food assistance programs like the Supplemental Nutrition Assistance Program (SNAP) shapes households’ risk of food insecurity [15–17]. However, few studies have specifically examined how rural families, which comprise a disproportionate share of SNAP recipients (14.6% compared to 11.5%) [18], access and interact with the federal food programs and policies that aim to mitigate the impacts of food insecurity.

Federal food assistance programs are one of the most important resources available to food-insecure families in the United States. SNAP is an especially significant resource for families living in poverty, particularly in light of cuts to traditional welfare programs during the Clinton Administration during the mid 1990s [17,19]. More than 40 million households receive SNAP benefits every month.[20] Studies have shown that SNAP helps reduce food insecurity, with one study demonstrating a reduction of 30% among SNAP recipients [21,22] However, not all families who need this type of food assistance are eligible, and SNAP benefits are insufficient to protect many households from the impacts of food scarcity [23–25]. Additionally, not all eligible recipients apply for SNAP and other forms of governmental assistance. Studies have identified multiple barriers to applying, including administrative burdens (e.g., long lines or cumbersome applications), a lack of information about how to apply, stigma, and fear of interacting with the government [4,13,26,27]. Comparisons of SNAP redemption rates in urban and rural areas demonstrate mixed results, with rural residents being less likely to participate than urban residents in some years and more likely in others [18,28].

People in both rural and urban areas experience stigma around using food assistance. Previous research demonstrates that stigma limits participation in SNAP and other social assistance programs and negatively affects recipients’ mental health [27,30,31]. One study found that more than half of all SNAP participants reported experiencing stigma, with no significant difference in the share of rural, suburban, and urban residents who experienced SNAP stigma [27]. Rural residents may experience distinct barriers to participation. For example, people in rural areas must travel farther to access SNAP offices and other social service providers [29]. People in rural areas may also perceive relying on public benefits as a moral failing that does not align with the “bootstrap” rural mentality that emphasizes self-reliance over dependence [14,26].

During the COVID-19 pandemic, expansions to SNAP and other food assistance programs lowered some of the barriers to access. For example, SNAP benefits were extended to maximum amounts for all recipients, and all children who qualified for free or reduced lunch received additional benefits through a new program called Pandemic-EBT (P-EBT). In North Carolina, where this study occurred, the P-EBT program provided SNAP benefits at a maximum of $370 per child over two installments the summer of 2020 to replace meals for families of students who would have received free or reduced price breakfast and lunches had schools been opened [32]. The program continued in 2021 and 2022. Most U.S. households also received stimulus checks from the government. As a result of interventions like these, after more than doubling at the beginning of the pandemic, rates of food insecurity fell back to pre-pandemic levels by the end of 2020 and stayed there in 2021. Since the expiration of many of these programs, food insecurity has again begun to rise [2]. Studies have shown how pandemic policies shaped households’ risk of food insecurity, but few have examined how these policies shaped people’s perceptions of food assistance and/or food insecurity [26,33].

Rural women’s perspectives are especially critical in food policy discussions, given that women are more likely to be the primary household food shoppers and live in households that are food insecure [2,12]. This is even more important when discussing food and health policies that impact families of color, as families of color are most likely to be food insecure (9.2% of Black families and 7.0% of Hispanic families compared to 4.0% of White families) [2]. Moreover, responsibility for preventing children from being food insecure falls most heavily on women, especially mothers [34]. Additionally, food, and in particular, the feeding of children, is often at the center of discussions about what it means to be a “good mother” [35,36]. For example, racialized concepts like the “good mother” and the “welfare queen” shape public discourse about food assistance and other health policies [36–38]. At the same time, some research finds that men are more likely to experience stigma around using SNAP, possibly because of gendered norms around food preparation and procurement [27]. These constructs around parenting, food, and health weave themselves into public consciousness, and as researchers have noted, social constructs in turn shape whether and how people interact with federal welfare policies and programs [39].

When it comes to understanding the role of government assistance policies in people’s lives, researchers have long adopted top down approaches that focus on policy makers and the decisions they make. As Michener et al. note, this provides an inadequate picture of government policy and the “inequalities it either produces, reproduces, or mitigates” [40]. Alternatively, they propose an approach to studying the welfare state from the perspective of those at its “margins.” This bottom-up approach centers the voices, experiences, and perspectives of people “who actually live with the consequences of elite decision-making” rather than just those making the decisions [40]. This is particularly important in rural communities. Researchers have documented how many rural residents feel neglected, disadvantaged, and disrespected by policies and politicians who don’t recognize the uniqueness of rural life [41–43]. Similarly, rural health scholars have called for more nuanced scholarship with rural communities—specifically, intersectional work that focuses on the “structural, historical, and policy influences” that shape disparate health outcomes in rural communities across markers of race, gender, class, and place [44].

In an increasingly fractured and polarizing political environment, understanding how people narrate and navigate the impacts of public health policy in their daily lives is critical to addressing issues like food insecurity, particularly in hard-to-reach rural areas. Although researchers have identified the significance of place in understanding political engagement [41–43], few studies have considered how people in rural communities think about health and food policy. Rural mothers’ perspectives, in particular, remain underrepresented in discussions and decisions over food assistance policies [45,46]. Therefore, this study adopted qualitative methodologies to identify and describe the food and health policy recommendations of low-income Black and White mothers in two rural counties in North Carolina, including what they wanted elected officials to know and understand about their lives and experiences of poverty and food insecurity. We propose that engaging the perspectives of diverse rural mothers living in poverty is critical to building common ground around U.S. food policy and provide recommendations for creating a more inclusive food assistance system that is centered around their narratives and experiences.

## Methods

### Community contexts

This study focused on two rural counties in North Carolina. North Carolina ranks second in the nation in terms of its rural population; almost 1 in 3 North Carolinians lives in a rural area, even as North Carolina’s urban areas are expanding at a rapid pace [47]. Both counties included in this study have significant Latinx populations (18.6% in one county, 10.7% in the second). Most of the population (approximately 60%) is White in both counties, with Black/African Americans comprising between 20% and 25% of the population in each. County rates of food insecurity were 15.7% and 16.4% at the start of our study.

### Data collection

This research is part of a broader longitudinal study about food access, poverty, and family life. In the larger study, researchers collected data between 2012 and 2020 with 124 female caregiver-child dyads in low-income households in three North Carolina counties. Recruitment for the study began on February 16, 2012 and ended on July 15, 2013. The study involved four waves of data collection that included interviews, surveys, and 24-hour dietary recalls. A team of Black, White, Latino/a/x, and Asian-American women and nonbinary researchers with varied class backgrounds conducted multiple semi-structured interviews and 24-hour dietary recalls with caregivers in years 1, 3, and 5 of the study. Of the original sample of 124 caregivers, 90% participated in Year 3 (n = 112), 73% participated in Year 5 (n = 90), and 44% participated in Year 8 (n = 54). Interviews in years 1, 3, and 5 took place in participants’ homes and lasted between 1.5 and 2 hours. In 2020 (Year 8), researchers conducted a brief (approximately 30−45 minutes) interview via Zoom with caregivers to learn about impacts of the COVID-19 pandemic on family food practices. Eligible participants were the primary caretaker (mother or grandmother) of at least one child between the ages of two and nine with household incomes at or under 200% of the poverty line when the study began. Caregivers provided written consent to participate in all years of the study, except for Year 8. Because interviews in Year 8 were conducted via Zoom during the early months of the COVID-19 pandemic, IRB approval was obtained for verbal consent during this year. Researchers audio recorded participants’ verbal consent for this interview. This research was approved by the Institutional Review Board at North Carolina State University.

This research study focuses specifically on caregivers living in the two rural NC counties of the study. The rural sample in year 1 included White (n = 41), Black (n = 24), and Latina participants (n = 20). This paper focuses on interviews conducted in two presidential election years: Year 5, which took place in Fall 2016 and Spring 2017 and Year 8, which took place in the second half of 2020. We limited this analysis to participants who were born in the United States and who were eligible to vote. Most of the Latina participants in the study were immigrants from Mexico or Central America. Many lacked legal documentation and were ineligible to vote in U.S. elections, and their fears of deportation shaped how they accessed food assistance and thought about the government [48]. The final sample included 50 total rural caregivers (n = 31 White, n = 18 Black, n = 1 Latina) in Year 5. The COVID-19 pandemic, which took place in year 8 of the study, limited our ability to connect with many participants from the study, making the sample size in Year 8 smaller than previous years. In Year 8, 32 rural caregivers (n = 21 White; n = 11 Black) were included in this analysis.

Interview questions across all waves of the study drew on existing work on gender, food, and parenting and asked about participants’ experiences related to feeding their families, including questions about food access, affordability, preferences, and the emotional and physical labor of food work [49]. In addition, in Years 5 and 8, we asked about what participants would like policymakers to know about their lives. These questions included how they felt about the presidential elections and what local, state, and federal government officials were doing well and what should change. Because we asked specifically about policymakers in Years 5 and 8, these two years are the focus of this study.

### Data analysis

During each year of the study, interviews were audio recorded and transcribed verbatim. The authors used pseudonyms for names and local places to protect participants’ identities. During each wave of the study, members of the research team developed a codebook and coded transcripts for key analytic concepts using NVivo software. A grounded theory approach, which pays close attention to how participants make meaning of their social worlds and attempts to generate theory from this lived experience, was used to develop codebooks each year, with a focus on caregivers’ food experiences and how their broader environment shaped choices and decisions around food [12,50]. The research team developed a codebook which and coded in two separate rounds. We kept codes broad so that we could carry out focused coding of these general concepts. Each year the coding process included reviewing 10% of transcripts, adjusting codes and recoding where necessary [51]. Reflexivity was a key component of the analysis. All members of the research team (n = 23) wrote reflective code memos, which focused on emerging insights related to data points, and allowed the research team to begin to tracking changes in perspectives over time [52]. In addition to writing memos, the research team shared and received feedback on research findings with participants via monthly newsletters, social media, and at community festivals. These reflexive practices allowed researchers to adjust protocols each year, learning from and with participants about emerging themes in the research that needed additional exploration and focus (for example, the impacts of elections on participants’ food practices and beliefs).

Qualitative longitudinal analysis was used to understand how caregivers thought about relationships with policymakers and elected officials over time. As part of this analysis, we created mini case profiles for each family, to understand how their food environment, practices, and circumstances changed over time [12,26]. We also coded data each year of the study and observed changes in themes over time. This paper relies on one code, created in Years 5 and 8, to capture discussions of elected officials, political administrations, or presidential elections. Based on this broad code, the authors identified themes within the data related to participants’ engagement with the 2016 and 2020 elections, the ways they framed impacts of new and potential health and food policy changes, and the ways their broader contexts shaped these reactions and responses over time. The authors read through coded data for each year and looked at how individual responses changed over time to understand the ways participants’ lived experiences and the broader historical environment shaped similarities and differences in this unique longitudinal data set [12,26].

## Results

Food insecurity fluctuated throughout the study (see Table 1). In Year 1 of the study, over half (53% n = 33) of rural families were classified as food insecure. 35% of rural caregivers (n = 17) were classified as food insecure according to the 10-item U.S. Adult Food Security Survey Module in Year 5. 28% (n = 9) were classified as food insecure in Year 8.

**Table 1:** Participant demographics.

	Year 5: 2016/2017 (n = 50)	Year 8: 2020 (n = 32)
Participant Race/Ethnicity		
*Black*	18 (36%)	11 (34%)
*Latina*	1 (.02%)	0
*White*	31 (62%)	21 (66%)
Food Security Status		
*Food Secure*	33 (66%)	23 (71.8%)
*Low Food Security*	9 (18%)	5 (15.6%)
*Very Low Food Security*	8 (16%)	4 (12.5)%)

Below, we summarize four main themes related to caregivers’ perceptions and experiences related to food policy and food assistance programs including: 1) ensuring children are fed, 2) increasing access and promoting dignity, 3) assessing deservingness of food assistance, and 4) recognizing maternal resilience. Caregivers’ feelings about food policy and U.S. elected officials differed across race/ethnicity, with White mothers reporting feeling disengaged from the political process and Black mothers expressing increased feelings of anxiety and worry about the impacts of elections and political events on their lives. White mothers were also more likely to embrace tropes of “deservingness,” questioning who should qualify for food assistance programs, when talking about the government’s spending priorities. Overall, caregivers interpreted politics through a lens of motherhood; they made recommendations based on perceived or potential impacts of policies and programs in terms of how they would impact their children (see Table 2).

**Table 2:** Representative quotes.

Theme	Representative Quotes
*“No Reason for Children to Go Hungry”*	“That they need to…not take away from those that are working and may not be able to cover everything and to allow people that may not have children to get food stamps and Medicaid and stuff like that.” Heather, White mother of two, Year 5 “That I think we need more food you know just for the kids for the kid’s sake you know I think there should be more lenient with it like parents single parents should get more for their kids to feed them, especially if they’re working and trying to do something more.” Shawna, Black mother of 3, Year 5 “So it’s like the gap of the cutoff between what they consider low poor income versus you’re over the minimum is I feel like they need to do something for those families that are right on the border, the ones that miss cutoff for assistance are the ones that really struggle because you have your poor economy that gets assistance, you know what I mean?” Tricia, White mother of 2, Year 8
*“They need to mandate”*	“Well I would tell them that it need to be where a poor class, and a middle class people could be able to eat just like the high class. God is so sick to see people digging in trash cans and stuff, and I heard but now I see it that people…can’t buy their medicine because it’s too high, and it’s a choice between eating and getting the medicine.” Sherry, Black grandmother of 2, Year 5 “It would be nice to definitely get healthier foods cheaper. Because I mean when you go into the grocery store it’s all the healthier stuff that is less sugar in it it’s so expensive so you can’t even afford it. Like you got to make sure you have food for the whole month so it’s like well if I buy this one little meal that costs it’s going to cost like three four days out of the you know my monthly planning.” Jenny, White mother of 4, Year 5 “I don’t think the food stamps are like the system for the, I guess people that are in a lower income is set up very well. Just trying somehow to get that set up better because it’s like I said when I’m working I don’t get any kind of assistance like that. That kind of makes it almost really hard to get ahead like I don’t think it’s set up where I don’t think it doesn’t seem like they want people to get ahead at all you know. It’s really kind of difficult.” Kelly, White mother of 2, Year 5 “Yeah, because with that and along with both of our incomes, I was able to get our credit card debt under control, so I was able to pay all that off and yeah, that really helped us. Yeah, I don’t know. I think we’re just pretty grateful of all the help we’ve had so far. I mean, there was a lot of people without jobs and they couldn’t get unemployment or anything, and both of our places were loyal to us and kept us employed, and it could have been a lot worse.” Jackie, White mother of 1, Year 8
*“We have our own people to take care of”*	“And then, another thing was the essential worker pay. These people who are unemployed are sitting at home getting extra unemployment, and the people that have worked throughout are still working and struggling.” Becky, White mother of 2, Year 8 “I stopped trying to fill it out at this point because we got denied a couple of years ago, and so I make a little bit more now too, so I know I’m not going to make it anymore. But back then it was, I’ve missed the cutoff by maybe $50 too much or something. And so we didn’t qualify. I am like 50 bucks? I mean, I pay $25 a week in school lunch. So that threshold where they count things off and on is a little, I think skewed, but we’re not starving, so we’re fine.” Tricia, White mother of 2, Year 8 “I have a comment on that, the stimulus check where they give it out. We could all use another stimulus check, but you have people that are getting stimulus checks for people that don’t even exist or they’re getting stimulus checks claiming kids that they don’t even have.” Clarissa, White grandmother of 3, Year 8
*“Only the strong will survive”*	“The only thing about the election is I believe that our state — our country doesn’t need a president. He is just someone who takes pictures. He is just someone who shakes hands.” Alix, White mother of 2, Year 5 “I haven’t paid any attention to none of it...Not really like I said I just go with the flow I deal with day by day, and that’s all you can do now these days.” Maria, White mother of 3, Year 5 “I definitely, I’m not in the political scene at all. I don’t know. I don’t really know enough about what’s going on to know what to say.” Beth, White mother of 2, Year 5 “Find out what’s going on because we’re sick of it. We can not make the country move forward if it’s a bunch of mess. Let’s get this mess out of the way. Don’t try to hold up for no one.” Winifred, Black Grandmother of 2, Year 5 “I just don’t want to raise mine no more and take everything I already what little bit I do get. Because he’s already slashing this and slashing that and take that no it’s going to be a rough four years. I pray he don’t make it for somebody that he did I’ve read somebody say let’s impeach him do something but um. We got to do better.” Georgia, Black mother of 2, Year 5 “We got to pay taxes on the end of the year anyway, so it doesn’t matter if we get it or not, they’re going to make us pay. They’re going to make us pay for it. I mean, yeah, it’d be great because it’d help me more money towards a house, but I’m just going to get taxed on it anyways. “Michelle, White mother of 2, Year 8 “I don’t know. Maybe they’ll send out another stimulus or try to help businesses some more. They’re going to have to do something. I mean, our economy is shocked, so they’re going to have to do something. I don’t know what, because I sure don’t have the answers” Stephanie, White mother of 2, Year 8

### *“No reason for children to go hungry”:* Ensure children are fed

Generally, caregivers felt that politicians didn’t understand what it was like to try to feed a family on a tight budget. Caregivers shared repeatedly that they wanted to purchase healthy foods—fresh fruits and vegetables, in particular—for their children, but the costs were too high. As Jordan, a White mother of 1, stated: “I just can’t afford it.” Several mothers talked about the structural inequities that shaped these realities. For example, Tricia, a White mother of 2, talked about the impacts of wage stagnation in her community: “That they, I mean, they, keep raising the prices, but they’re not raising what people are making… So it’s making it harder to get the same amount of food that you normally would.” Even when pandemic policies led to an increase in the food assistance that mothers received, it remained a struggle to feed everyone. Specifically, mothers described how it had become harder to feed everyone due to the combination of having children home all day during stay-at-home orders, not always being able to access resources at food pantries and at school, and experiencing disrupted supply chains. Mothers wanted politicians to care about their struggles and provide more support for people who were doing all they could to get by. When asked what she wanted government officials to know about families and food, Kyla, a Black mother of 3 shared, “It’s no reason, it’s no excuse for children to go hungry.”

Caregivers emphasized how hard they worked to keep their children fed. They felt this work went unseen by those with political power. Many also felt penalized even when they tried to “get ahead.” For example, mothers explained that when they started working more hours or earned a higher paying job, this could put them “right at the border” of losing access to SNAP benefits. Shawna, a Black mother of 3, shared: “I think we need more food for the kid’s sake. I think [the government] should be more lenient with [SNAP]. Like parents, single parents should get more for their kids to feed them, especially if they’re working and trying to do something more.” Caregivers called on governmental officials to recognize the work they did to keep children fed and address the penalties they faced when they tried to get by.

### *“They need to mandate”:* Increase access and promote dignity

Caregivers called on elected officials to create programs that made it easier to access the foods and resources they needed in a way that protected their human dignity. They wanted elected officials to experience what it was like to stand in line at a food pantry for hours only to be handed “rotten” foods. They also wanted them to understand the difficult trade-offs they were making with their incomes, like the “choice between eating and getting…medicine” (Sherry, Black grandmother of 2). They felt stigmatized by having to seek out help and instead wanted elected officials to support programs that centered human dignity and were easy to access. In 2016, Pam, a White mother of 2, shared:

A mother ought to be able to say, ‘Well baby, if you want that, you go right ahead and eat that.’ That’s what I’d tell [policymakers] they need to mandate… Don’t make people think that they’re begging for help. That’s exactly what these places do, they act like that you’re begging them to help you… I’m sitting here begging you, and you’re still going to deny it. My young’un’s hungry; she don’t understand that I can’t go to the store and get her nothing to eat.

Like Pam, other caregivers described how they wanted to protect their children from the indignities of poverty and food insecurity. They also wanted the social service systems they interacted with to promote and protect dignity as well, instead of making people feel that they are “begging” for help.

The COVID-19 pandemic provided the types of “mandates” that Pam had called for during her interview. The U.S. implemented several policies that were applied universally. For example, all SNAP recipients began receiving the maximum level of benefits, and the P-EBT program offered additional benefits for all families whose children qualified for free and reduced meals. Several mothers shared how these additional funds enabled them to “catch up,” and see a bit of financial hope for their future, a theme that caregivers rarely reported prior to the pandemic. Jackie, a married White mother of 1, shared that “with [the stimulus check] and along with both of our incomes, I was able to get our credit card debt under control… and that really helped us.” Kimberly, a Black mother of 1, echoed this, saying the stimulus check allowed her to “have time to think and catch up on things…So I just feel like it is just a breather.” Immediate and accessible support like this was important because mothers didn’t have to “beg” to get help from the government or other financial aid programs. Instead, they shared how it provided some much-needed “breathing room” from their regular financial and food struggles.

### “*We have our own people to take care of*”: Focus on deservingness

Although caregivers advocated for accessible support that promoted dignity, many expressed concerns about people who they felt did not deserve assistance. These concerns were often racialized, with White caregivers more likely to use language of deservingness to talk about public assistance. For example, when reflecting on the 2016 election, Clarissa, a White grandmother of 3, shared, “We have too many immigrants in here [the U.S.] right now. We have our own people to take care of. We have homeless veterans.” Whereas Clarissa focused on immigrants as the people who she felt did not deserve help, others expressed a broader but related concern about making sure U.S. tax dollars supported U.S. families and not people in other countries. Becky, a White mother of 2, shared, “We can’t save the world if we can’t save us. We have so many…that are hungry and starving…but yet we’re trying to fund billions of dollars to different countries to help them out when we can’t even help ourselves.”

Some people judged specific people in their community when talking about deservingness. Several White caregivers discussed how they’d seen public benefits being used by people who “don’t need [benefits, because they] can really work” (Janice, White mother of 3). During the COVID-19 pandemic, some mothers expressed resentment that they continued to work in hazardous or unsafe conditions while others were getting unemployment benefits. As Miranda, a White mother of 2, shared, “I kind of feel like we’re being slapped in the face. We’re putting ourselves out there just like the doctors, the nurses, the paramedics, the cops. And we’re not seeing any extra money anywhere.”

Caregivers evoked a language of deservingness in complicated and sometimes contradictory ways. Some pushed back against their perception that other people were judging them as undeserving. At the same time, across markers of race and identity and across years of the study, many caregivers didn’t see *themselves* as deserving of public benefits or emergency food assistance. Tricia, a White mother of 2, for example, had visited a food pantry at the onset of the pandemic, but stopped after a few months, sharing, “We feel like there’s people that could use it more, who are more in need… It’s not like our pantry’s dry.”

### “*Only the strong will survive*”: Recognize maternal resilience

Caregivers expressed how they would “make a way” to make sure their families were fed because they didn’t always trust that the government would support them. They felt that politicians didn’t always care about the people whose food security was at stake. As Alberta, a Black mother of 3 shared, politicians are “affecting people’s lives.” Reflecting on what they saw as confusion and chaos in the early days of the first Trump administration, several caregivers reflected that they hoped elected officials would instead offer concrete solutions to address poverty and hunger. In both 2016/7 and 2020, caregivers expressed anxiety about potential cuts to federal food assistance programs. These programs were often discussed by politicians during the process of reauthorizing the 2018 Farm Bill and on the 2020 presidential campaign trail. As Kitty, a White mother of 3, shared in 2017, “I’ve heard some rumors where they want to take food stamps and stuff like that away. I hope not, because it will bring struggle to my family.”

Many caregivers worried about potential cuts to food assistance programs. However, there were differences by race in how they thought about how politics shaped their day-to-day lives. Several White caregivers told us they hadn’t voted in 2016 because they felt their vote didn’t matter. For some, this was because politicians “always tell you what you want to hear, and when they get in, it’s totally different what they do” (Kitty, White mother of 3). As Samantha, a White mother of 3, shared, “I’m not into politics. I mean he [the President] doesn’t affect my life or my children’s life.” By contrast, many Black mothers did feel that the President influenced their lives. Several drew on faith when articulating how they would get by if federal benefits were drastically cut or programs were eliminated altogether. Katina, a Black mother of 2, feared cuts to federal programs but shared, “I really think that all the big talk is just… I don’t know; I’m praying that he’ll [the President will] have a change of heart.” Georgia, a Black mother of 2, also prayed for a change, but reiterated that her faith was active in the face of struggle:

I’m hoping it works out, and I guess we just need to really start making our own businesses and being able to take care of ourselves because they ain’t nobody else going to do it…We got to start building up our communities ourself because there ain’t nobody else going to do it. We don’t get [it] - they ain’t going to care.

In general, Black mothers articulated a form of moral agency that emphasized faith and maternal resilience in the face of what they saw as the potentially devastating impacts of national elections.

Despite these racialized differences, most caregivers emphasized their own agency when describing how they would face potential program cuts or keep children fed during a global pandemic. Carletta, a Black mother of two, found ways to stretch dollars in the face of food shortages in 2020. She shared, “We ration what we have. You know–-I make sure the kids eat first and we have meals that we can stretch.” Despite fearing that things were “going to get worse” before they got better, caregivers prioritized feeding their children and said they would do whatever it took to make sure their children had enough to eat. As Beatrice, a Black mother of 1, put it, “Only the strong will survive.”

## Discussion

This research builds on other studies of health policy in the rural United States [11,13,53,54] by examining how rural women caregivers think about food policy in light of experiences of poverty and, for some, food insecurity. These narratives call attention to the diversity of rural communities and the complex ways rural caregivers make sense of their political landscape as they work to ensure their children are fed. In popular and public health research, rural areas tend to be described as deficit-laden areas; however, rural communities are also “nuanced, thriving” spaces with critical assets and people who are working to cultivate health and well-being [44]. As Afifi et al write, “Understanding the complexity of rural is critical to the development of more responsive social and health policy and services that promote health equity” [11]. This type of nuanced and complex assessment is particularly important given the marginalization of rural women’s voices and perspectives in rural health work and research [9].

Previous research shows that experiences of food insecurity and participation in food assistance programs are both associated with negative mental health outcomes, in part due to stigma as well as experiences of disenfranchisement that make it difficult to utilize these programs [27,55]. Caregivers in this study similarly described challenges to participating in federal food programs. They drew on these experiences to recommend policies to better support families, make it easier to apply for and receive services, and address gaps in programmatic support. For example, caregivers described the ways that that their jobs and wages often fluctuated, which resulted in reductions in their SNAP benefits, leading to increased struggles in feeding their families despite a higher income or new job. Caregivers in this and other studies have recommended changes to the SNAP program that reduce experiences of stigma and disenfranchisement, including increasing benefits so that parents can buy healthier foods for their children and updating program eligibility requirements to allow families “on the border” of receiving SNAP benefits to still qualify for support when they take a higher paying job [13,56]. This aligns with other research that has documented how working families experience gaps in SNAP eligibility [13,53,56] and other forms of disenfranchisement that keeps parents from accessing food assistance programs [15,26].

Previous research demonstrates how participants in food assistance programs often feel stigmatized and shamed when applying for, and utilizing benefits to pay for food and beverages at authorized stores [27,55]. Stigma shapes whether and how people access services, and impacts their health in turn [27,30]. Even among people who utilize SNAP, researchers have found that experiences of stigma are associated with reported food insecurity and poor mental health outcomes [27,31]. Caregivers in this study noted similar feelings of mental distress around how to feed their families and narrated how experiences of stigma shaped their use of food services and programs. Caregivers advocated for expanding food assistance programs to promote human dignity and keep parents from feeling like they must “beg” for services. Although limited research exists on this topic, principles of universal design can be harnessed to decrease experiences of stigmatization and prevent parents from feeling that they have to, “beg” for help. Universal programs also increase participation in SNAP and other federal food assistance programs, promoting better ease of use, clarifying eligibility, and increasing accessibility [57–59]. For example, the social safety net expansions that occurred during the COVID-19 pandemic led to decreases in food insecurity and poverty from 2020 to 2021 [16]. More than $1 billion in funding for federal food assistance programs was passed in 2020, which created the P-EBT program for families qualifying for free and reduced-price school meals and expanded SNAP. Since these programs have been repealed or scaled back, rates of food insecurity have again increased [2], suggesting that more generous and more universal food policies can mitigate food insecurity [60].

The notion of “deservingness,” expressed in particular by some White caregivers, offers insight into one potential challenge of expanding benefits in rural areas. Several of the White mothers in this study talked about deservingness as a moral concept, rooted in a strong sense of individualism and self-determination. This individualism not only judges others as “lazy” or undeserving of benefits, it also reflects the conflicting relationship that many rural White Americans have with safety net programs they often participate in [41,61]. Race is often a lens through which policy is interpreted by both the public and policy makers [62]. For example, Maltby and Kreitzer found that Black survey respondents were more likely than White respondents to see users of social welfare programs as deserving; by contrast, White respondents were only as likely as Black respondents to do so when they knew someone impacted by those social welfare policies [39]. Concepts like deservingness, which impact whether and how people are eligible for and access government services, must be analyzed with close attention to both race and place.

Our study shows how racialized experiences and assumptions shape rural caregivers’ understandings of the role of elected officials in their lives. Several White mothers disengaged altogether from the political process, feeling that elected officials didn’t impact their lives. Black mothers did not have the privilege of disinvestment. Instead, they had a clear-eyed view of how elected officials shaped their lives, often drawing on faith and their experiences as mothers to illumine what Curtis et al. have named as caregivers’ sense of “moral agency” in the face of oppression and hardship [63]. Black caregivers recognized, and often feared, the power that political and social actors played in their lives. However, in the face of dehumanizing rhetoric and proposed policy changes that threatened their family’s well-being, they also reiterated that they would “make a way” and care for their children, even if it meant leaving the U.S. This form of moral agency reflects what Hotz has described as a “subversive” form of agency that historically marginalized groups often adopt in the face of “destructive social messages about their worthiness” [64].

Across all markers of race, caregivers didn’t see elected officials as reliable or consistent, echoing other research on rural consciousness that demonstrates how many rural communities feel they are ignored by politicians and left to fend for themselves [41]. These experiences are exacerbated for families living in poverty, who have often been systematically excluded from political processes that have concentrated wealth and resources in the hand of a few and increased poverty in the U.S [65]. Caregivers likewise recognized that it was their responsibility to ensure their children were fed, even if that meant jeopardizing their own health. This resonates with other research demonstrating that mothers often sacrifice their own health to mitigate the impacts of food insecurity on their children [12,15,26,66]. Related, many mothers throughout our study didn’t see themselves as deserving of public support and services; they felt others were worse off. While drawing on their personal and community strengths demonstrates resilience in the face of disinvestment, the individualism embedded in this form of rural consciousness can also work against efforts to adopt more inclusive and supportive policies for all [54,55]. By contrast, government officials in Oregon have worked to humanize the issue of food insecurity by reminding the public of the “real people” (often hardworking families with children) behind hunger statistics, echoing the recommendations of caregivers in this study. Researchers have noted that these efforts to empathize with people, rather than shame or blame them, have cultivated a more compassionate political environment that has played a role in more public support for social safety net programs and increased enrollment in vital services like SNAP [67].

## Limitations

There are several limitations to this study. First, the perspectives come from rural caregivers in two counties in North Carolina. Their experiences and narratives are distinct to their context and community and may not reflect the views of rural caregivers elsewhere. Additionally, we did not include the perspectives of non-U.S. born caregivers, because their engagement with political leaders was distinct compared to other mothers. As rural areas become increasingly racially and ethnically diverse [68], additional analysis is needed to understand how immigrant mothers navigate similar anxieties about feeding their children within a backdrop of deportation fears and increased anti-immigrant sentiment. The research also does not include the perspectives of other caregivers in the family; additional research is needed to understand how fathers and other care providers feel about policy and policy makers as it relates to food. Because of the small sample size, we were not able to explore intergenerational dynamics, particularly among the few grandparents raising grandchildren. Additional research could explore how these intersectional and generational dynamics impact families’ experiences of food insecurity. Finally, the 2020 interviews reflect the beginning of the pandemic; additional research is needed to understand how families adjusted to changes in safety net policies, as well as how rural people’s perspectives on poverty, food insecurity, and food assistance continue to evolve.

## Conclusions

To build consensus and move toward inclusive and just health policies in an increasingly fraught political climate, we must be attentive to the nuances of how people navigate the impacts of elections and elected officials on their everyday lives. As others have noted, rural women are marked by an “intersectional invisibility” that has kept their perspectives and voices out of public policy discourse [9]. Mothers bear the burden of child-rearing, are overwhelmingly responsible for feeding children, and disproportionately feel the emotional and mental load of raising children. Many balance this while working outside the home [12,15,66]. Parents—and mothers in particular—are often left out of conversations about political representation and health policy, despite the fact that families act “as an important agent for transmitting civic and political skills across generations” [69]. Policy solutions to entrenched problems like food insecurity need to reflect the complexities and nuances of rural spaces by centering the diverse voices of the caregivers who live there.

Engaging caregivers in the democratic process, particularly those living in poverty and in rural areas, will mean building more inclusive practices that recognize and address these realities, and cultivating systems that invite their full participation. This will take creativity and care. Building common ground on federal food policy should center these marginalized voices, drawing on intersectional praxis to learn with (rather than about) rural caregivers and paying close attention to how they interpret health policy within their contexts and communities. This type of community-based research should aim for long-term engagement to understand changes in communities over time and build relationships that engender trust and empower communities to reflect on their own narratives and experiences to envision a positive future for the places and people they love.
